# The World Bacterial Biogeography and Biodiversity through Databases: A Case Study of NCBI Nucleotide Database and GBIF Database

**DOI:** 10.1155/2013/240175

**Published:** 2013-10-21

**Authors:** Okba Selama, Phillip James, Farida Nateche, Elizabeth M. H. Wellington, Hocine Hacène

**Affiliations:** ^1^Microbiology Group, Laboratory of Cellular and Molecular Biology, Faculty of Biological Sciences, USTHB, BP 32, EL ALIA, Bab Ezzouar, Algiers, Algeria; ^2^Environmental Microbiology, School of Life Sciences, University of Warwick, Coventry CV4 7AL, UK

## Abstract

Databases are an essential tool and resource within the field of bioinformatics. The primary aim of this study was to generate an overview of global bacterial biodiversity and biogeography using available data from the two largest public online databases, NCBI Nucleotide and GBIF. The secondary aim was to highlight the contribution each geographic area has to each database. The basis for data analysis of this study was the metadata provided by both databases, mainly, the taxonomy and the geographical area origin of isolation of the microorganism (record). These were directly obtained from GBIF through the online interface, while *E-utilities* and *Python* were used in combination with a programmatic web service access to obtain data from the NCBI Nucleotide Database. Results indicate that the American continent, and more specifically the USA, is the top contributor, while Africa and Antarctica are less well represented. This highlights the imbalance of exploration within these areas rather than any reduction in biodiversity. This study describes a novel approach to generating global scale patterns of bacterial biodiversity and biogeography and indicates that the *Proteobacteria* are the most abundant and widely distributed phylum within both databases.

## 1. Introduction

Biogeography aims to explain spatial patterns of diversity in the context of evolutionary events such as speciation, dispersal, extinction, and species interactions [1]. Macroecologists have long studied the biogeography of higher plants and animals in various habitats [2, 3]. In contrast, there is very little information available on the biogeography of prokaryotes. This stemmed from the difficulty of assessing microbial communities by cultivation methods, which only sampled 0.1% to 10% of the microbial community [4]. However, with the advent of cultivation-independent sequencing techniques, microbial communities of many environments have been characterized, including soil [5], the Arctic and Antarctic Oceans [6], and the Sargasso Sea [7]. This, in turn, facilitated prokaryotic biogeography studies in a number of environments on scales ranging from 0.002 km to 20,000 km [1] and from scale of a nation [8] to intercontinental scale [9].

Data from many of these biodiversity studies are stored in databases, a structured and organized collection of information where the storage of and the access to information are facilitated to users. In biosciences, the introduction of computer processing and computer databases has opened up the potential for further investigation of combined existing data sets [10]. These include the study of specie distributions through both time and space and their use as an educational resource (both formal and public), for conservation and scientific research, use in medicine and forensic studies, in natural resource management and climate change, in art, history, and recreation, and for social and political use. Uses are many and varied and may well form the basis of much of what we do as people every day [11]. 

In our study, we used the concept of species occurrence data, mainly, observational data, and environmental survey data. In general, the data are what we term “point based,” although line (transect data from environmental surveys, collections along a river), polygon (observations from within a defined area such as a national park), and grid data (observations or survey records from a regular grid) are also included. The majority of point-based data used here are georeferenced; that is, records with geographic references tie them to a particular place in space—whether with a georeferenced coordinate (e.g., latitude and longitude, UTM) or not (textual description of a locality, altitude, depth)—and time (date, time of day). Often, the data are also tied to a taxonomic name, but unidentified collections may also be included [12]. We retrieved bacterial records for different worldwide geographical areas, countries/islands, which were stored in NCBI Nucleotide Database and GBIF Database [13, 14] and then assigned them to their respective phyla. This was in order to describe the world bacterial biogeography at a broad taxonomic scale in terms of taxa proportional abundance by contributed records from each geographic region. Since databases are growing fast, we limited our search to a determined period, data published on/before December 25, 2012.

## 2. Material and Methods

### 2.1. Hardware

One personal computer was used having a Dual Core CPU E5800 @ 3.20 GHz processor and 2 GB RAM. Internet connection was tested as 1.36 Mbps download and 5.55 Mbps upload [15].

### 2.2. The Approach

The approach used in this study for both databases is divided into three parts:

- database query→
- data subset retrieval (bacterial records verifying the query structure) in standardized response format for each geographical area→
-  analyze data and save the information summary for each geographical area.

#### 2.2.1. Databases

*GBIF Database*. The Global Biodiversity Information Facility (GBIF) was established as a global megascience initiative to address one of the great challenges of the 21st century—harnessing knowledge of the Earth's biological diversity. GBIF envisions a world in which biodiversity information is freely and universally available for science, society, and a sustainable future. GBIF's mission is to be the foremost global resource for biodiversity information and engender smart solutions for environmental and human well-being. At the time of writing, the GBIF Database include 396,026,747 records, 345,561,101 of which have associated georeference data (March 3, 2013 at 10:32) (Version 1.2.6) [10–13].

*The NCBI Nucleotide Database*. The National Center of Biotechnology Information (NCBI) Nucleotide Database is a public database along with 52 others that belong to The National Center of Biotechnology Information (NCBI), which is a division of the National Library of Medicine (NLM) at National Institutes of Health (NIH). The database is formed of a collection of nucleotide sequences from several sources, including GenBank, which is part of the International Nucleotide Sequence Database Collaboration (INSDC), which is comprised of the DNA DataBank of Japan (DDBJ), the European Molecular Biology Laboratory (EMBL), and GenBank at NCBI. These three organizations exchange data on a daily basis—the NCBI Nucleotide Database also includes sequences from NCBI Reference Sequences (RefSeq), Third Party Annotation (TPA), and from Protein Data Bank (PDB). At the time of writing, the NCBI Nucleotide Database included 78,756,144 records (March 3, 2013 at 04:30) [14]. 

#### 2.2.2. List of Geographical Areas

The list of geographical areas used in this study was obtained from the International Nucleotide Sequence Database Collaboration (INSDC) through controlled vocabulary for “/country qualifier” [16]. The study also included the distribution of bacteria among the seven continents.

#### 2.2.3. List of Phyla

Common phyla were selected from the NCBI Taxonomy (number of species: 11,364 with 31 phyla) [17, 18] and the catalogue of life taxonomic classification (number of species: 9,072 with 25 phyla) [19], used respectively by NCBI Nucleotide and GBIF databases. The final list included 24 common phyla, listed as follows: 

bacteria main groups = *[“Acidobacteria”, “Actinobacteria”, “Aquificae”, “Bacteroidetes”, “Chlamydiae”, “Chlorobi”, “Chloroflexi”, “Chrysiogenetes”, “Cyanobacteria”, “Deferribacteres”, “Deinococcus-Thermus”, “Dictyoglomi”, “Fibrobacteres”, “Firmicutes”, “Fusobacteria”, “Gemmatimonadetes”, “Lentisphaerae”, “Nitrospirae”, “Planctomycetes”, “Proteobacteria”, “Spirochaetes”, “Thermodesulfobacteria”, “Thermotogae”, “Verrucomicrobia”]*.

#### 2.2.4. Access Databases

*GBIF Database*. The number of records with geographic coordinates from the GBIF Database is displayed through the GBIF species portal [20]. The bacterial records were retrieved from GBIF Database for each of the geographical areas of the study through the occurrence search webpage. The keywords used in “Add search filter” were “Bacteria” for the Taxonomy (Scientific Name) filter and the respective “geographical area's name” for the Geospatial filter. The generated results were downloaded as spreadsheet zipped files [21]. Once downloaded, a *Python* script (version 2.7.3) [22] (see Supplementary Materials: GBIF_Filter.py available online at http://dx.doi.org/10.1155/2013/240175) was used to filter files and to retrieve the occurrences of bacterial records for each geographical area based on a simple algorithm (see Algorithm 1: Biodiversity and Biogeography—GBIF_Filter). 

*NCBI Nucleotide Database*. The general way (simple, direct, and manual) to query NCBI Nucleotide Database (save/extract data) is by using web services through a web browser [14]. However, this method is not adapted for automatic multitask queries—that is, for the search of information about few organisms, the user has to introduce queries, one by one, for each organism and to retrieve records each time. Thus, the search would be time consuming, and for a large number of organisms would be manually impossible. Similarly to the two other INSDC partners, EMBL and DDBJ, NCBI provides a programmatic access to various data resources and analysis tools via web services technologies. 

*Programmatic Retrieval System for NCBI Nucleotide Database Records*. The programmatic access for NCBI records passes through the Entrez Programming Utilities (NCBI *E-utilities*), a set of eight server-side programs that provide a stable interface into the Entrez query and database system at the NCBI [23] and a computer language. In this study, *Python* (version 2.7.3) was used with *Biopython* package (version 1.60) [22, 24]. First, *Python* posts an E-utility URL to NCBI and then retrieves the results of this request, after which it processes the data as required [23].

When using the geographical area's name directly as a search term, for instance “France”, the results retrieved would give all sequences where the word “France” is mentioned. This is problematic as, for example, results returned would include those where authors institutions are in France rather than the country of origin of the sample, which is required. 

A new qualifier has been added since December 15, 1998; this is about the “qualifier/country”, which would “restrict” the search to records that include the geographical origin of the sequence [16].

Using the word “country” or “/country” as an additional word for the search will restrict the search. Yet, similar problems are encountered when using records generated from collaborative international work. The result would include overlap records since “country” is considered as an ordinary word, and the standard search in this case would be for every researchable field for the combination of both the geographical area's names and the word “country” without distinguishing between the origin of the sequence and the collaborating country(ies). To verify this, using an additional name of a geographical area, for instance “Italy”, in the query structure of the search as “country France Italy”, will result in giving overestimated records where both countries are mentioned although the sequences are registered to only one geographical area.

As there is no direct method to access the “qualifier/country” by a simple query structure, and to be more restrictive and more accurate, additional computer processing to return the desired sample location using the “qualifier/country” should be applied.

For each of the retrieved records, where the “geographical area's name” and the word “country” were used as keywords for the filter, we extract the whole information value included in the “qualifier/country” field when it exists [16]. Then, for each record, we match the information to the geographical area's name of interest; if it matches, we count the record and we consider its phylum.

A *Python* script was written; see supplementary materials: NCBI_Nucleotide_Tracker.py, based on an algorithm (Algorithm 2: Biodiversity and Biogeography—NCBI_Nucleotide_Tracker) which encompasses three main parts as below.

1. Define the query structure:
  - the query structure: “*country* AND *geographical area's name* AND *Bacteria[Organism]* AND *date of publication*”
    - *country*: to limit the search to records that may have the qualifier/country;
    - *geographical are producing active biomolecule a's name*: to precise the geographical area in the search, and this with respect to the INSDC list;
    - *Bacteria[Organism]*: to limit the search to bacteria domain;
    - *date of publication*: to limit the search to a time period;
    - AND: Boolean operator, the intersection, used to narrow the search results to the joint part of the subset results of the other words in the query.
2. Connect the script to the NCBI Nucleotide Database: query the database and retrieve the data as a standard format (GenBank format, so the real qualifier/country can be accessed), and this is mainly handled by *Biopython* package.
3. Analyze data: filter the data, access the “qualifier/country”, and match the qualifier value to the searched geographical area's name of interest; if it matches and then the record is counted and the taxonomy is recorded. Finally, the summary of this analysis for each geographical area is saved.

Since the computer processing used here is word processing, particular geographic areas were analyzed independently, differentiating certain ambiguities; for instance, “Republic of the Congo” and “Democratic Republic of Congo” are different countries but both contain “Republic of Congo” within the qualifier. A third *Python* script, modified from the previous NCBI_Nucleotide_Tracker.py, was used in combination with an exception list to circumvent this problem, (see supplementary materials: NCBI_Nucleotide_Exception.py) results are registered in a file (see exception.txt supplementary materials).

#### 2.2.5. The World Biogeography Maps

Data from this study was used to generate world bacterial biogeography maps. The package “*rworldmap*”, available on CRAN, was used for the mapping and visualization of global data working under the environment “*R language-version 2.15.1*” [25, 26].

## 3. Results

### 3.1. General Queries

#### 3.1.1. GBIF Database

The occurrences overview for records with coordinates for the seven kingdoms of life, extracted from GBIF Database through the GBIF Species Portal, is summarized in Table 1. It is clear from the results that *Eukaryota*, mostly animals and plants with nearly 95%, are the dominant registered records, whereas bacteria represent less than 0.5% of all records.

#### 3.1.2. The NCBI Nucleotide

Data in Table 2 show the results for general queries using different filters through the NCBI Nucleotide Database webpage. GenBank is the most used database to register sequences compared with the INSDC partners (DDJB) and (EMBL). We also observed that most records were found to be nucleotide sequences of *Eukaryota* 64%, while bacteria represent just nearly 10%. Additionally, from the 72,020,824 records found in the NCBI Nucleotide Database, only 17% as 11,994,306 would be tied to a particular geographical area. 

### 3.2. Bacterial Biogeography and Biodiversity

While the INSDC's list contains 275 geographical areas and an additional 12 historical country names, the final list of this study includes only 208 common geographical areas. This was either because some geographical areas do not appear in both databases, for example, Borneo and Taiwan or there were no bacterial records for these in the GBIF Database, for example, Bahrain, Swaziland, and Jersey.

From the 208 geographical areas of this study, for the GBIF Database, using filters as described above, and after downloading files, 1,222,216 records were recovered. In total, using the Catalogue of Life Taxonomic Classification, 88% of all retrieved records were assigned to one of the 24 phyla common with NCBI Taxonomy; see supplementary materials: gbif_Classification_2000_Plus.txt and NCBI_GBIF_overall_data.xlsx.

Conversely, using the programmatic access approach to query the NCBI Nucleotide Database, we could retrieve information on 3,232,147 records which satisfied the query structure with: the name of the geographical area, the word “country”, and bacteria as organism, of those which were assigned to the right geographical area was 2,322,339, 56% −1,311,049 of those which were assigned to one of the 24 phyla common to Catalogue of Life Taxonomic Classification. Moreover, 1,233,118 records were retrieved as environmental samples in NCBI Nucleotide Database using this method. These could also be environmental samples within already-assigned phyla see supplementary materials: country_all.txt and NCBI_GBIF_overall_data.xlsx. 

#### 3.2.1. The Relative Abundance of Different Phyla

**Figure 1:**
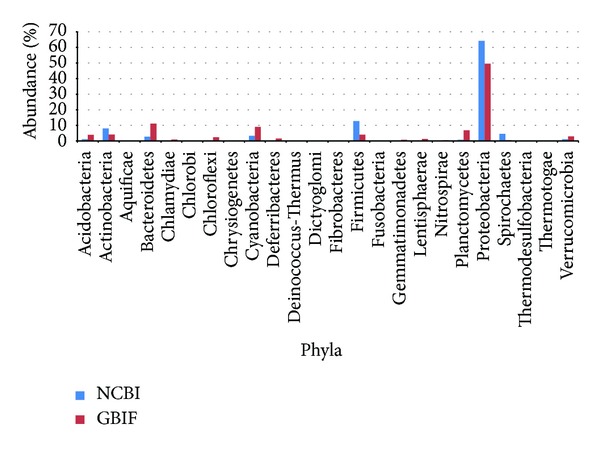
The relative abundance of the 24 common phyla in NCBI Nucleotide Database and GBIF Database.

Records retrieved from both NCBI Nucleotide and GBIF databases summarized in Figure 1 and Table 3 show that *Proteobacteria* are the most abundant phylum in both databases with 64% and 49%, respectively, *Firmicutes* 13% and *Actinobacteria* (8%) were the second most abundant phyla for NCBI Nucleotide Database, and *Bacteroidetes* (11%) and then *Cyanobacteria* (9%) and *Planctomycetes* (7%) for GBIF Database. The remaining phyla represented less than 5% each. In the last position, we may find *Chrysiogenetes* and *Dictyoglomi* with less than 0,004% of records for both databases.

#### 3.2.2. Overall Geographical Occurrences of Different Phyla

Records retrieved from both databases summarized in Table 3 show that the most distributed phylum was *Proteobacteria*, covering 83% of records for GBIF Database and 90% for NCBI Nucleotide Database for all geographical areas in this study. *Actinobacteria*, *Cyanobacteria*, and *Firmicutes* had more than 50% coverage each in both databases. *Bacteroidetes* distribution seems to be more important using data from NCBI Nucleotide Database 50% than data from GBIF Database 36%. Eleven phyla had a similar degree of distribution among the two databases with less than 5% difference in terms of record numbers. A difference between databases in terms of phyla global distribution was noted for the *Acidobacteria*, *Chloroflexi*, *Plactomycetes* and *Spirochaetes*, which were more widely distributed in the NCBI Nucleotide database, while *Deferribacteres*, *Fibrobacteres*, *Fusobacteria*, and *Lentisphaerae* were more widely distributed in the GBIF database. Those with less than 5% of coverage and coming from less than 10 geographical areas in both databases were the* Thermodesulfobacteria*, *Dictyoglomi* and *Chrysiogenetes* which are considered to be really restricted to certain geographical areas.

Finally, considering GBIF Database alone, we also observe that 12 of the 24 phyla were distributed with nearly 20% coverage for the whole 208 geographical areas nearly 40 geographical areas.

#### 3.2.3. Occurrences of Records in Different Geographical Areas

**Figure 2:**
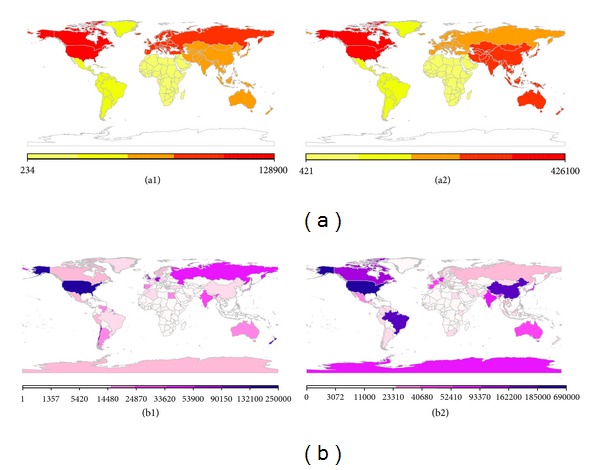
The world biogeography (a) by continent in (a1). GBIF Database. (a2). NCBI Nucleotide Database. (b) By country in (b1). GBIF Database and (b2). NCBI Nucleotide Database.

Table 4 shows the occurrences of records by continent for both NCBI Nucleotide and GBIF databases. The American continent has the largest number of records submitted, representing 39% of all registered records in GBIF Database and more than 50% in the NCBI Nucleotide Database, yet only half 634,225 of these NCBI Nucleotide records are assigned to one of the 24 phyla. Europe with 27% and Australia-Oceania with 16% are second and third, respectively, for the contribution of the GBIF data input, while Asia is more likely to contribute records in the NCBI Nucleotide Database with 21%, ranking second than to the GBIF Database 11%. Antarctica is less involved with 1% and 4% of the world bacterial biodiversity being registered for GBIF or NCBI Nucleotide databases, respectively. Finally, there is nearly 3% of data registration from Africa in each database. The world maps for bacterial biogeography regarding continents are illustrated in Figures 2(a1) and 2(a2). 

For a close look at the top ten countries for both NCBI Nucleotide and GBIF databases recovered records and their assignment to the 24 phyla, Table 5 reveals that USA occupies the first place for both databases. The number of records from GBIF would be greater than this since the GBIF maximum records number returned per file is 250,000. Two countries, Germany and India, ranked in this list for both databases. For the rest of the geographical areas, we observed different patterns for the two databases. The world maps for bacterial biogeography regarding countries are presented in Figures 2(b1) and 2(b2). 

We also observed from Table 5 that while the continents and the top ten countries bacterial records occurrences assignments were close to the overall assignment average (88%) for the GBIF Database, the continents and the top ten countries assignments vary enormously from the average assignment (57%) of NCBI Nucleotide Database. 

## 4. Discussion

The study reveals that most bacterial biodiversity was retrieved from developed countries and USA, particularly. The bias seen in these databases toward developed countries may be attributed to several reasons: these countries encompass technological platforms, especially, for the massive of both sequencing and registration of data and are engaged in a number of biodiversity exploration projects, and yet the most important reason is research and development funding budget. To maintain its position as a world leader in science and research, USA has invested a huge budget over the two last decades, and this is continuously increasing. The forecast for the 2014 USA budget is $142.8 billion; it calls for a federal basic and applied research investment totaling $68.1 billion, up to $4.8 billion or 7.5 percent increase compared to the 2012 enacted level [27]. On the other hand, less biodiversity is observed in many areas, particularly countries in Africa and in Asia (the Middle East and Central Asia); we do not suggest that less real biodiversity is present in these countries, but rather that less microbial biodiversity targeted research is performed, and thus less of the generated data are submitted to the different databases. 

While we could retrieve information on 3,232,147 records from the NCBI Nucleotide Database as they satisfy the query structure, it is obvious that if compared with a simple general query used through the NCBI Nucleotide Database website as “Country AND Bacteria”, we would notice a difference of additional 955,219 records. This may be explained, as stated before, by the overestimation of records. Moreover, the registered records do not reflect the exact number of strains isolated or observed in a geographical area, since it is possible to find many sequences belonging to the same strain, for a redundancy or the fact that they are fragments of one genome (example: *Streptomyces globisporus* C-1027 from China is registered as 557 times for whole genome shotgun sequencing).

Forces shaping the biogeography of macroorganisms—including dispersal limitations, habitat differentiation, competition, and adaptive radiation—have been a central focus of ecology for more than a century [28]. Yet, while microorganisms are the most abundant and diverse organisms on Earth [29], relatively little is known about the patterns of, or controls over, microbial distribution within and between the planet's major habitat types. One common theory holds that the tremendous dispersal potential of microbes will lead to everything being everywhere (i.e., no dispersal limitations), with environmental selection determining which species are abundant [1]. However, until recently, methodological limitations have prevented large-scale tests of ideas about where certain microorganisms exist and why [30, 31].

Over the last decades, however, molecular phylogenetic approaches have revolutionized microbiology, expanding our view of microbial diversity and our appreciation of the complexity of microbial communities [30]. While these techniques do not provide an exhaustive sampling of any but the simplest microbial assemblages, they do provide information on the dominant members of the community, allowing ecologically meaningful questions to be addressed about the distribution of these lineages. These methods have been used to reveal that some microorganisms exhibit distinct biogeographical patterns [1, 32, 33] and are demonstrated to be the vast majority [34] which appear to be controlled by differences in environmental variables in some cases [32], and geographical distance in others [35, 36], while the few abundant organisms were more likely to be widely distributed [34], and those may form a common diversity structure within soil bacterial communities around the globe [37]. Other works investigating overall community composition support the role of environmental gradients in structuring both lake and soil bacterial communities [38, 39]. Biotic interactions may also be important in determining microbial community composition; a recent study showed that microbial communities exhibit more segregation of taxa than would be predicted by chance, suggesting that competitive interactions and/or niche specialization may be important in structuring bacterial biogeography [40]. Similar to Nemergut et al. [34] and within our study of both databases, although it only involved the phylum rather than the inferior taxonomy ranks, we have shown that the abundant phyla (*Proteobacteria*, *Actinobacteria*, *Cyanobacteria*, *Firmicutes*, and* Bacteroidetes*) are the most distributed, whereas the majority of less abundant taxa are predominantly located in particular regions. Yet, these results have to be taken with care especially for geographical regions where few records are registered which would not reflect the bacterial diversity within those regions.

In terms of data quality, the collector and then the submitter of the record(s) have the primary responsibility for data quality in both databases [12]. While the submission of record(s) is possible by anyone to NCBI, the GBIF accepts only credited organisms already registered and approved by the latter. In our study, we have found that NCBI Nucleotide Database seems to cover a larger area and would be the only available resource for bacterial diversity in some regions, for instance, Andorra, Bahrain, and Equatorial Guinea. However, it is more likely to be influenced by the biomedical research policy of the leading country and its National Institutes of Health (NIH) this observation is not only toward this database but also toward many of the generated data in several research projects of life sciences; this may be also understood when we examine the annual budget that has been invested in research and development awarded to the National Institutes of Health (NIH) which was of $30 billion for the year 2012. This was nearly half of the expenditure for the nondefense R & D budget [14, 28], so it is obvious to see a certain preference for the exploration and the registration of a particular category of microorganisms than others, for example, microorganisms interfering with health, inducing diseases, or producing active biomolecules (antibiotics, antitumoral …).

While the queries were submitted on November 25, 2012, submitting the same queries and readying this paper would generate slightly different results, and this is due to the update process for both databases.

## 5. Conclusion

New technological advances and approaches are emerging from sampling to data analysis, and this is to cope with the diversity and complexity of life. Therefore, data generated in biosciences are growing exponentially. Analysis software and methods must also keep up with this rapidly expanding field so that the most can be made of current studies within this field. It is unknown how the patterns that we observed today may change with the upcoming “daily results”; our study is considered to be the first attempt to catch the first snapshot of a particular moment on the world bacterial biogeography and biodiversity through the usage of both NCBI Nucleotide and GBIF databases. 

Despite these constraints, our approach may be extended to other domains of life (*Archaea*, *Eukaryota*) or even for a more restrictive group of taxa (example: *Actinobacteria *and all subtaxa within this group).

For the NCBI Nucleotide Database, the same approach could generate more information on the retrieved sequence, such as: length, type DNA or ARN, single sequence, complete genome or shotgun sequencing, and function of the gene: 16S RNA gene or other genes. Almost all information from any qualifier of a record would be extractable, which may answer some of the questions that we may ask: who is doing what? How and why study these strains? Is it perhaps for producing active biomolecules (antibiotics, antitumor …), or for diversity studies, and so forth, and this would be possible by adding few lines regarding the qualifier in need. 

Moreover, we suggest that the registration of information regarding the qualifier “/country” should be obligatory. Again, as it has been mentioned by NCBI Nucleotide, it has to be clear for the submitter that this qualifier is to indicate the origin of the sequence. The geographical area's name indicated by the INSDC should be respected when registering or searching for data. We also suggest that regions have to be defined to avoid ambiguity with a different format, for example: uppercase, or put in another field. Besides, the search for the qualifier “/country” should be facilitated by simple search word structure, for instance, CountryName[country] as applied for other search qualifiers, for example: OrganismName[Organism] for organisms. The methodology used in this study would also retrieve the diversity in particular regions within a geographical area of interest either by declaring it as previously described or adding it as a subcondition after the search. While the new qualifier “/lat_lon available as 2005”, which indicates the GPS coordinates for the location at which a specimen, from which the sequence was obtained, was collected, it would be very useful and more accurate to determine the strain origin. This biogeography search for a particular region is much easier in GBIF, simply by either using bounding box or introducing coordinates (latitude, longitude, altitude, and depth) in the occurrence webpage as filters. 

It would be also possible and interesting to associate this biogeography study to ecological keywords which would highly be recommended to be completed by users. This association would be used in biodiversity informatics which surely generates worthwhile knowledge not only about the presence of the microorganism but also about its probable involvement in the ecosystem function and its different interactions.

One big challenge to the comparison of different databases is to cope with many different standards: for the registration and the retrieval system, data structure, and even the differences on fundamental aspects such as in taxonomical classification which was one example encountered in our study; where phyla: *Synergistetes, Caldiserica, Elusimicrobia, Armatimonadetes, Ignavibacteria, Tenericutes, Thermomicrobia, *and the newly established *Nitrospinae* phylum are considered either different or completely absent in one or another database used in this study. All of these points and others are more and more being discussed worldwide by the scientific community [17, 41].

While the web interface is easier to deal with databases, the programmatic access seems to be more interesting, more flexible, offers more choices, and returns more personalized results; however, it needs some basic knowledge on the database structure, its database management system, and computer languages.

Finally, while the study gives a preliminary overview of the world's bacterial biogeography, reflecting a part of the real biodiversity, other more upcoming efforts to determine Earth microbial biogeography and biodiversity are indeed in progress, we could mention “Earth Microbiome Project”. The project already processed over 200,000 samples from across the globe for these microbial communities using metagenomics, metatranscriptomics, and amplicon sequencing and started to generate huge amount of data to produce a global Gene Atlas describing protein space, environmental metabolic models for each biome, approximately 500,000 reconstructed microbial genomes, a global metabolic model, and a data-analysis portal for visualization of all information [42].

## Supplementary Material

The zipped file may be extracted in any directory in the computer but all files and the two folders should be extracted in the same directory so the scripts would function correctly. The scripts had been tested on Windows environment. Python 2.7 and Biopython 1.6 should be installed before running the scripts. To extract data from NCBI Nucleotide you should run “NCBI_Nucleotide_Tracker.py” for countries exception handling, run “NCBI_Nucleotide_Exception.py”. For the GBIF Database, only three countries are provided as an example in the folder “GBIF_Plus” for additional countries you may download them from GBIF website, the only data that should be extracted are these of taxonomy, no need for other metadata (Dataset, Geospatial) as described in the paper. Once all countries of interest are downloaded you may run “GBIF_Filter.py” to filter data. The results of this study are presented in: “country_all.txt” for NCBI Nucleotide Database, “gbif_Classification_2000_Plus.txt”, “absent_taxa_Classification_2000_Plus.txt”, and absent_taxa_Classification_2000_Plus_ex_All.txt” for GBIF Database. The final results are presented in “NCBI_GBIF_overall_data.xlsx”. Some errors that may occur during the script execution are presented in “error.txt” file.

## Figures and Tables

**Algorithm 1:**
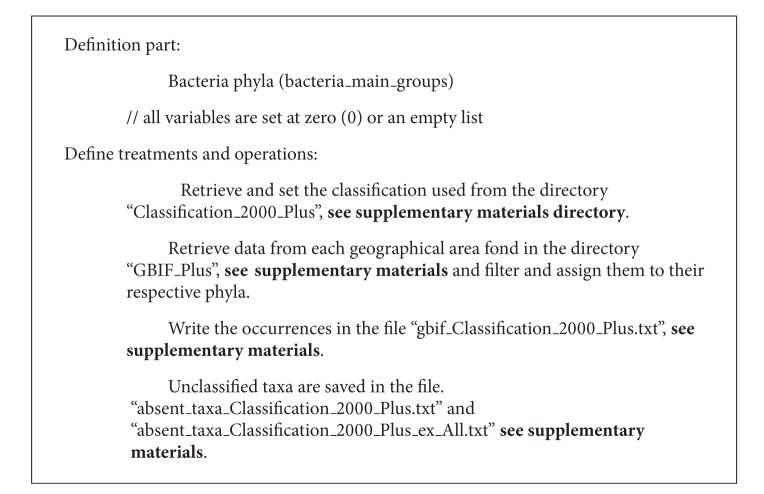
Biodiversity and biogeography—GBIF_Filter.

**Algorithm 2:**
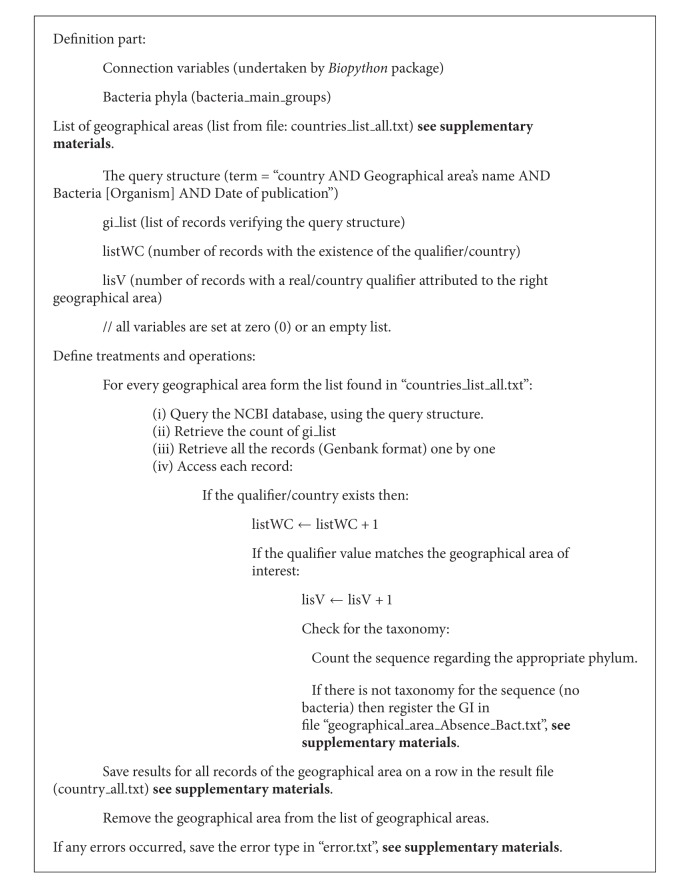
Biodiversity and Biogeography—NCBI_Nucleotide_Tracker.

**Table 1:** Occurrences overview of records with coordinates from GBIF Database.

Kingdom	Link	Records with coordinates	Percentages
Archaea	http://data.gbif.org/species/2	26,501	0,008
Bacteria	http://data.gbif.org/species/3	1,593,278	0,479
Animalia	http://data.gbif.org/species/1	238,944,036	71,785
Chromista	http://data.gbif.org/species/4	1,539,408	0,462
Fungi	http://data.gbif.org/species/5	6,176,944	1,856
Plantae	http://data.gbif.org/species/6	80,044,950	24,048
Protozoa	http://data.gbif.org/species/7	3,916,926	1,177
Incertae sedis	http://data.gbif.org/species/0	616,822	0,185

**Table 2:** Results of general queries using different filters through the NCBI Nucleotide Database webpage.

Query	Records
Search “all” [Filter] Limits: Published between: 1986/1/1 and 2012/11/25	72,020,824
Search “ddbj” [Filter] Limits: Published between: 1986/1/1 and 2012/11/25	10,323,758
Search “embl” [Filter] Limits: Published between: 1986/1/1 and 2012/11/25	11,259,765
Search “genbank” [Filter] Limits: Published between: 1986/1/1 and 2012/11/25	44,146,674
Search Bacteria [organism] Limits: Published between: 1986/1/1 and 2012/11/25	7,156,037
Search Archaea [organism] Limits: Published between: 1986/1/1 and 2012/11/25	306,675
Search Eukaryota [organism] Limits: Published between: 1986/1/1 and 2012/11/25	46,489,750
Search “country” Limits: Published between: 1986/1/1 and 2012/11/25	11,994,306
Search ((country AND Bacteria [organism])) Limits: Published between: 1986/1/1 and 2012/11/25	2,276,928
Search (((country AND Archaea [organism]))) Limits: Published between: 1986/1/1 and 2012/11/25	130,882
Search (((country AND Eukaryota [organism]))) Limits: Published between: 1986/1/1 and 2012/11/25	8,346,238

*The Nucleotide Advanced Search Builder was used to construct the queries.

**Table 3:** The relative abundance and the overall geographical occurrences of the 24 common phyla in NCBI Nucleotide Database and GBIF Database.

	Acidobacteria	Actinobacteria	Aquificae	Bacteroidetes	Chlamydiae	Chlorobi	Chloroflexi	Chrysiogenetes	Cyanobacteria	Deferribacteres	Deinococcus-Thermus	Dictyoglomi	Fibrobacteres	Firmicutes	Fusobacteria	Gemmatimonadetes	Lentisphaerae	Nitrospirae	Planctomycetes	Proteobacteria	Spirochaetes	Thermodesulfobacteria	Thermotogae	Verrucomicrobia
NCBI																								
Ab	15043	106127	1417	35795	1489	858	4762	9	43896	152	710	37	821	167616	375	1819	79	3118	9734	841254	59943	80	381	15534
%	1,147	8,095	0,108	2,730	0,114	0,065	0,363	0,001	3,348	0,012	0,054	0,003	0,063	12,785	0,029	0,139	0,006	0,238	0,742	64,166	4,572	0,006	0,029	1,185
GBIF																								
Ab	65711	75156	688	193454	14418	3799	46328	2	167900	26883	2201	4	1889	71480	2375	12896	22999	8337	117391	841535	11087	211	272	48806
%	3,897	4,146	0,032	11,136	0,899	0,202	2,446	0,000	8,999	1,600	0,124	0,000	0,102	3,967	0,132	0,766	1,303	0,464	6,848	49,449	0,583	0,010	0,013	2,878
NCBI																								
Oc	55	144	20	112	37	32	62	5	114	21	47	6	16	139	26	39	14	40	64	189	92	9	21	56
%	26,442	69,231	9,615	53,846	17,788	15,385	29,808	2,404	54,808	10,096	22,596	2,885	7,692	66,827	12,500	18,750	6,731	19,231	30,769	90,865	44,231	4,327	10,096	26,923
GBIF																								
Oc	42	124	14	75	41	35	44	1	177	40	40	2	28	112	40	41	41	41	46	173	47	8	29	47
%	20,192	59,615	6,731	36,058	19,712	16,827	21,154	0,481	85,096	19,231	19,231	0,962	13,462	53,846	19,231	19,712	19,712	19,712	22,115	83,173	22,596	3,846	13,942	22,596

Oc: the overall geographical occurrence of a phylum was calculated as the occurrence of at least one record per geographical area. Ab: relative abundance of phyla.

**Table 4:** Occurrences of records by continent for both NCBI Nucleotide and GBIF databases.

Continents	GBIF	%	Assigned	% assigned	NCBI	%	Assigned	% assigned
AMERICA	481976	39.435	421526	87,458	1200669	51.701	634225	52,823
AFRICA	42289	3.460	37972	89,792	55796	2.403	39723	71,193
EUROPE	335373	27.440	306014	91,246	371561	15.999	214725	57,790
ASIA	143984	11.781	126967	88,181	504874	21.740	341823	67,705
AUSTRALIA-OCEANIA	204615	16.741	182665	89,273	96073	4.137	72257	75,211
ANTARCTICA	13979	1.144	13363	95,593	93366	4.020	8296	8,885

Total	1222216		1088507		2322339		1311049	

**Table 5:** Top ten countries list for NCBI Nucleotide and GBIF databases recovered records and their assignment to the 24 phyla.

Countries	Records (GBIF)	Assigned %	Countries	Records (NCBI)	Assigned %
USA	250000	89.224	USA	689988	60.753
New Zealand	132127	87.822	China	185045	66.324
United Kingdom	88823	95.639	Brazil	173997	57.799
Germany	90153	83.569	India	82663	89.801
Chile	84339	82.525	Germany	74444	67.535
Netherlands	53903	94.308	Mexico	40678	84.972
Russia	49308	83.563	Japan	87861	39.219
Northern Mariana Islands	33624	90.519	Australia	48788	66.400
Portugal	30651	93.064	Spain	48057	62.861
India	31981	88.840	France	52411	56.563

## Abbreviations

NCBI: National Center of Biotechnology Information
GBIF: Global Biodiversity Information Facility.
